# Self-Adaption of the GIDL Erase Promotes Stacking More Layers in 3D NAND Flash

**DOI:** 10.3390/mi14030686

**Published:** 2023-03-20

**Authors:** Tao Yang, Bao Zhang, Qi Wang, Lei Jin, Zhiliang Xia

**Affiliations:** 1 Institute of Microelectronics of the Chinese Academy of Sciences, Beijing 100029, China; 2 University of Chinese Academy of Sciences, Beijing 100049, China

**Keywords:** 3D NAND Flash, GIDL erase, self-adaption, drain-to-body potential

## Abstract

The bit density is generally increased by stacking more layers in 3D NAND Flash. Gate-induced drain leakage (GIDL) erase is a critical enabler in the future development of 3D NAND Flash. The relationship between the drain-to-body potential (*V*_db_) of GIDL transistors and the increasing number of layers was studied to explain the reason for the self-adaption of the GIDL erase. The dynamics controlled by the drain-to-body and drain-to-gate potential contribute to the self-adaption of the GIDL erase. Increasing the number of layers leads to a longer duration of the maximum value of *V*_db_ (*V*_db_max_), combined with the increased drain-to-gate potential, which enhances the GIDL current and further boosts channel potential to reach the same value at different positions of the NAND string. We proposed a method based on the correlation between the duration of *V*_db_max_ and the number of layers to obtain the limited layers of the GIDL erase. The limited layers allowed are more than four times the number of layers used in the current simulation. Combining the novel method of dividing the channel into multi-regions with the asynchronous GIDL erase method will be useful for further stacking more layers in 3D NAND Flash.

## 1. Introduction

With the continuous development of smartphones, 5G, artificial intelligence, and cloud computing, the demand for higher bit density in the market has grown rapidly. The bit density is generally increased by stacking more layers in 3D NAND Flash [1,2,3,4,5,6,7,8,9]. However, the connection scheme of using a body contact spacer (BCS) between channel polysilicon and the array common source line faces challenges during the process of removing the bottom of the gate stack, especially when stacking more than two stacks due to the overlap problem [10]. The novel connection scheme of the channel-hole sidewall ONO butting (CSOB) scheme was proposed to boost the storage density by using the sidewall connection scheme between channel polysilicon and the array common source line [10]. The erase method was changed from body erase in the BCS scheme to GIDL erase in the CSOB scheme. Therefore, holes needed to be generated by gate-induced drain leakage (GIDL) to increase the channel potential and achieve the erase operation. The first challenge for GIDL erase is the control of the doping profile in GIDL transistors, especially when it is first used in Bit Cost Scalable (BiCS). Since the manufacturing process of the bottom select gate is formed prior to the memory cell, the memory cell requires processes with a high thermal budget to achieve a large memory window and better performance. However, this leads to the problem of doping in the bottom select gate, as it can spread from the heavily doped source diffusion during the high thermal budget process [11]. To solve this problem, the manufacturing process of the bottom select gate was changed in Pipe-BiCS, placing it after the memory cell and adopting a shallower and steeper select gate channel doping profile with the low thermal budget [12]. Since then, the GIDL erase has faced challenges related to the variability and uniformity of the erase potential due to the indirect bias of GIDL erase caused by the potential drop for band-to-band tunneling. Caillat et al. proposed an optimization method for GIDL erase with dual-side (bottom select gate side and top select gate side) GIDL injection, which led to better erase effectiveness and more effective tail control of the erase threshold voltage distribution for variability improvement [13]. Malavena et al. studied the GIDL erase dynamics process in the vertical channel NAND Flash, focusing on the increase in channel potential, and proposed a compact model for the dynamic process of the GIDL erase to explore the directions for continuous optimization [14,15]. Nowadays, most of the advanced manufactured 3D NAND Flash structures in production use the GIDL erase method. The industry expects that the cost-effective manufacturing of 3D NAND Flash can achieve over 1000 stack layers via innovations in processes and novel materials [16]. Meanwhile, combining these innovative solutions with file-system and firmware-level optimizations can greatly improve memory performance and expand the application market of 3D NAND Flash memory [17]. GIDL erase is a critical enabler for the future development of 3D NAND Flash. However, the challenge of GIDL erase is the limitation layer, as more holes are required to boost the channel potential with stacking more layers. This has become a potential risk for the realization of thousands of layers in 3D NAND Flash.

In this work, we have investigated the correlation between GIDL erase and the number of layers (*N*_Layers_) based on TCAD simulation. The relationship between the drain-to-body potential (*V*_db_) and *N*_Layers_ was studied. We found self-adaption of the GIDL current (*I*_gidl_) with an increase in the *N*_Layers_. As the *N*_Layers_ increased, the duration of the maximum value of *V*_db_ (*V*_db_max_) also increased, resulting in a longer duration of maximum GIDL current. The self-adaption of the GIDL erase is achieved through the dynamic control by the drain-to-body and drain-to-gate potential. At the end of the duration of *V*_db_max_, which leads to maximum *I*_gidl_, the same potential can be reached at different positions of the NAND string. The proposed method enables the determination of the limited number of layers for the GIDL erase based on the correlation between the duration of *V*_db_max_ and the number of layers. Finally, we propose to divide the channel into multi-regions and implement an asynchronous GIDL erase method to overcome the limitation of GIDL erase and enable the stacking of more layers in 3D NAND Flash.

## 2. Simulations

**Figure 1:**
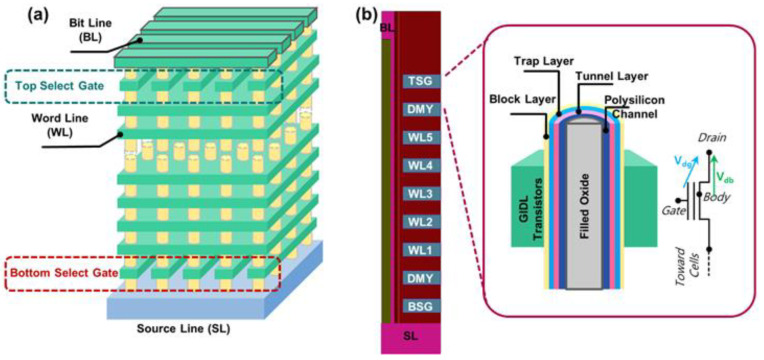
(**a**) Schematic diagram of 3D NAND Flash structure; (**b**) schematic diagram of TCAD simulation structure: the inset shows the detailed structure of the GIDL transistor, and the gate stack consists of the tunnel layer, trap layer, and block layer, respectively.

Figure 1a shows the schematic of the 3D NAND Flash structure. The Synopsys Sentaurus Sdevice simulator (Synopsys, Mountain View, CA, USA) was used for the simulation. The simulation structure of this device is shown in Figure 1b, with one top select gate (TSG), one bottom select gate (BSG), and more than two dummy cells (DMY) between the select gate and memory cells, and the number of memory cells will be dynamically adjusted according to the *N*_Layers_. The TSG and BSG function as GIDL transistors. The parameters of the simulation structure are based on the current technology products. Furthermore, the drift–diffusion model, Coulomb scattering mobility model, thermionic emission, Shockley–Read–Hall recombination, and band-to-band-tunneling (BTBT) model were introduced in the polysilicon channel, which was well-calibrated based on our previous works [18] and GIDL current experiments. The inset shows the detailed structure of the GIDL transistors. The BTBT current occurs in the virtual PN junction around the GIDL transistors, and it follows the channel conduction direction through the BTBT path. Meanwhile, the electric field in the virtual PN junction is controlled by the drain-to-gate potential and the drain-to-body potential. GIDL transistors at the top and bottom of the strings can be applied as a negative bias to the gate relative to the drain. The erase voltage can be transferred to the channel via hole injection generated by the BTBT during the erase operation.

## 3. Results and Discussion

**Figure 2:**
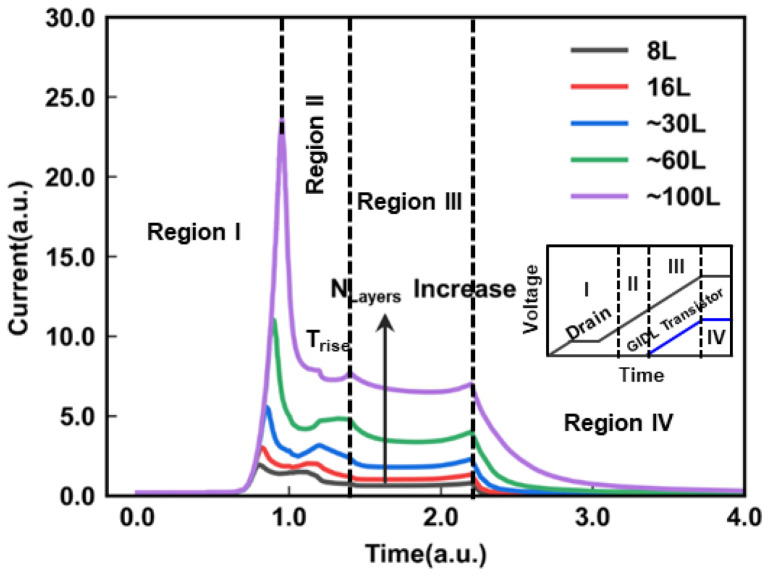
The *I*_gidl_ with the increasing *N*_Layers_: the inset shows the waveform of the GIDL erase operation.

**Figure 3:**
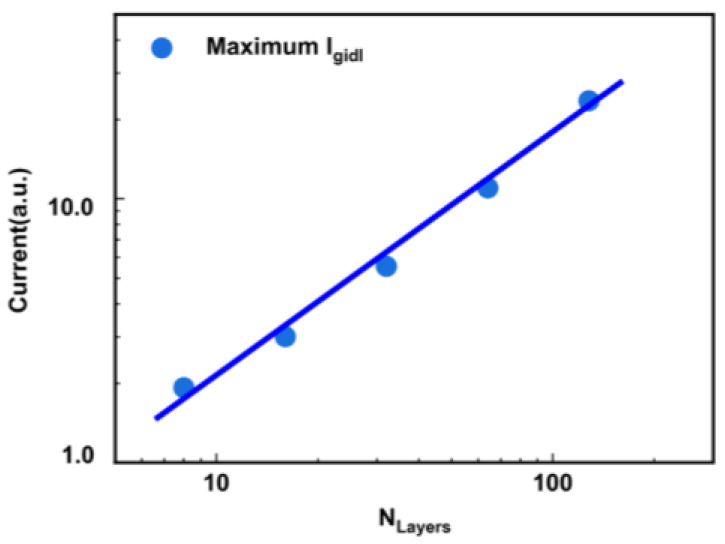
The correlation between the maximum value of the *I*_gidl_ and the *N*_Layers_ extracted using Sentaurus TCAD.

Figure 2 shows the GIDL current during the GIDL erase operation for various *N*_Layers_. There is a significant dependence of the *I*_gidl_ on the *N*_Layers_, which can be divided into four regions. Region I is before *I*_gidl_ reaches the maximum value. Region II is before the voltage of GIDL transistors starts to increase (*T*_rise_), Region III is before the end time of the ramp-up of the drain voltage, and Region IV is the main erase region with constant erase voltage. In Region I, the maximum *I*_gidl_ value for larger *N*_Layers_ occurs later than the maximum value for small *N*_Layers_. As the *N*_Layers_ increase, there is a time delay in the appearance of the maximum value of the *I*_gidl_. Once the *I*_gidl_ reaches the maximum value, it rapidly begins to decrease. In Region II, the larger *N*_Layers_ exhibit a steeper decrease in the *I*_gidl_ from the maximum value. In Region III, the *I*_gidl_ remains relatively constant after the voltage of the GIDL transistors starts to increase. The larger *N*_Layers_ result in a higher *I*_gidl_. In Region IV, the *I*_gidl_ also decreases rapidly, which shows an obvious dependence on the *N*_Layers_. Finally, the GIDL current tends to be the same value regardless of the *N*_Layers_ in the main erase region. The reason why the *I*_gidl_ depends on the number of layers is that the capacitance of the channel increases as more layers are stacked, requiring more holes to be injected to boost the channel potential for GIDL erase. Then, we extracted the maximum value of the *I*_gidl_ for each of the *N*_Layers_. As shown in Figure 3, there is a clear correlation between the maximum value of *I*_gidl_ and the *N*_Layers_ in a log scale. The *I*_gidl_ generated by the BTBT has increasing self-adaption with an increase in the *N*_Layers_.

**Figure 4:**
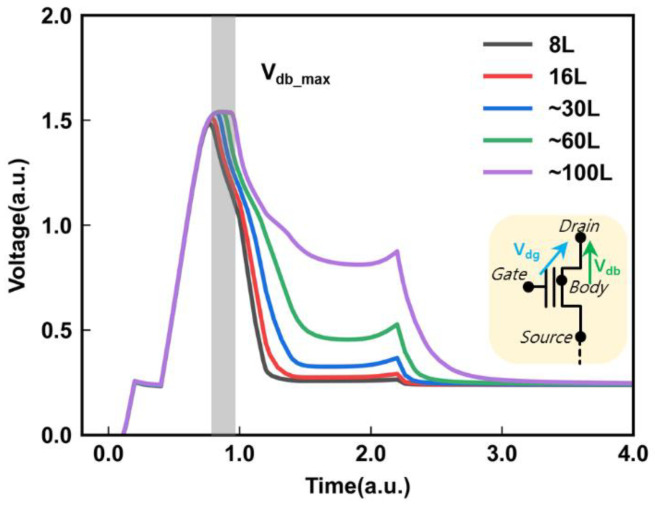
The drain-to-body potential with increasing *N*_Layers_, and large *N*_Layers_ with long *V*_db_max_ time.

**Figure 5:**
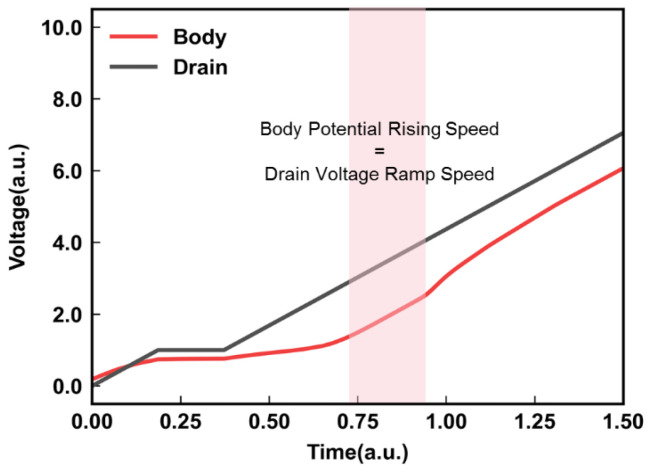
The correlation between drain and body potential.

The electric field that drives the BTBT in the junction is controlled both by the drain-to-gate potential (*V_dg_*) and the drain-to-body potential (*V*_db_). In the experimental test, the *V*_dg_ can be easily monitored. However, monitoring the changes in the drain-to-body potential is difficult. To investigate the reason for the self-adaption of *I*_gidl_ with increasing *N*_Layers_, we used Sentaurus TCAD simulation tools to study the relationship between *V*_db_ and the *N*_Layers_. Figure 4 shows the dependence of *V*_db_ on the *N*_Layers_. Before *V*_db_ reaches the maximum value (*V*_db_max_), the trend of *V*_db_ is independent of the *N*_Layers_. This is due to the small *I*_gidl_, resulting in a slower rise in the channel potential in the body area after the hole injection compared to the ramp speed of the drain voltage. However, once *V*_db_ reaches *V*_db_max_, the duration of *V*_db_max_ exhibits a clear dependence on the *N*_Layers_. The increase in the *N*_Layers_ leads to a longer duration of *V*_db_max_. The appearance of *V*_db_max_ is attributed to the equal rise rate of the GIDL transistors’ body potential and the external drain voltage ramp speed (shown in Figure 5), which allows *V*_db_ to remain in the *V*_db_max_ state. This can be explained by the fact that the capacitance of the channel increases with an increase in the *N*_Layers_, which requires a larger *I*_gidl_ to affect the far-end channel potential and needs more time to meet the demand of *I*_gidl_. Therefore, keeping *V*_db_ at *V*_db_max_ between the drain-to-body and *V*_dg_ will create a further increase, promoting a significant increase in the GIDL current to meet the demand for higher layers.

**Figure 6:**
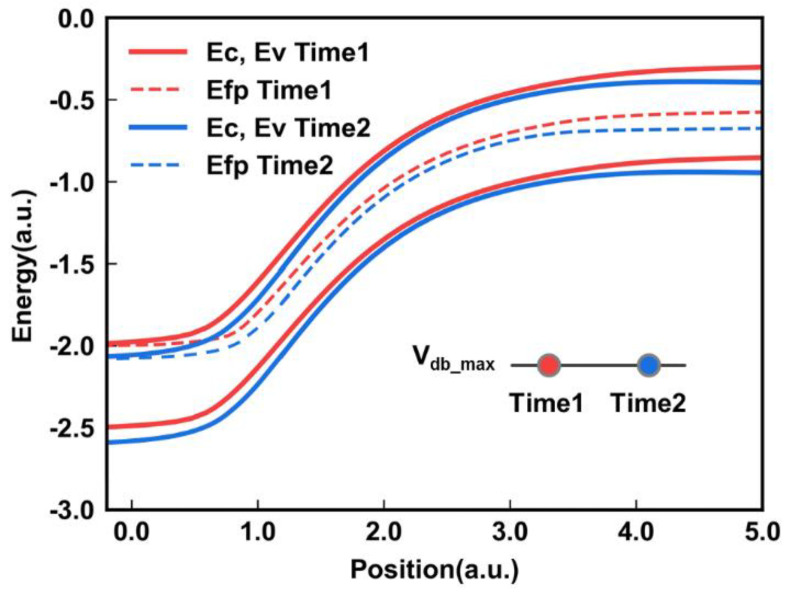
The band diagram of GIDL transistors at various times with the *V*_db_max_, Time2 > Time1.

Figure 6 illustrates the band diagram at various times with the *V*_db_max_. With an increase in time, a larger *V*_dg_ reduces the BTBT distance, leading to a higher *I*_gidl_. At the final time of *V*_db_max_, *I*_gidl_ reaches its maximum value. After that, *V*_db_ starts to decrease sharply from the maximum value, and this region is closely related to the *N*_Layers_. The final *V*_db_ increases with the number of layers. At this time, the *V*_dg_ remains constant, and the junction is affected by *V*_db_ to dynamically generate *I*_gidl_ to meet the demand for a higher number of layers. The dynamics controlled by the drain-to-body and drain-to-gate potential contribute to the self-adaption of the GIDL erase, which enables one to stack more layers in 3D NAND Flash.

**Figure 7:**
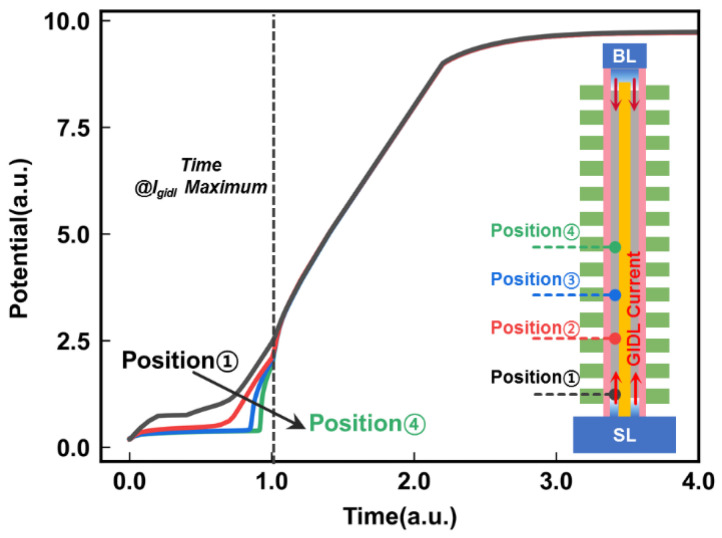
The channel potential at different positions of string during the GIDL erase operation.

Next, the channel potential at different positions of the string during the GIDL erase operation was studied, as shown in Figure 7. Malavena et al. assumed that the channel potential remains as a constant channel potential due to uniform hole accumulation [15]. To investigate the channel potential position dependence of the GIDL erase, we used a verified channel length longer than 5 um. However, our results show an interesting phenomenon. There is an apparent channel position dependence on the channel potential before the GIDL current reaches its maximum value. When the GIDL current reaches the maximum value, the channel potential starts to reach the same potential, and *I*_gidl_ becomes large enough to boost the total channel potential. Then, the channel potential and the position potential of the GIDL transistors become the same. It is clear that the *I*_gidl_ reaches the maximum value during the GIDL erase process, and the same potential can be reached at different positions along the NAND string.

**Figure 8:**
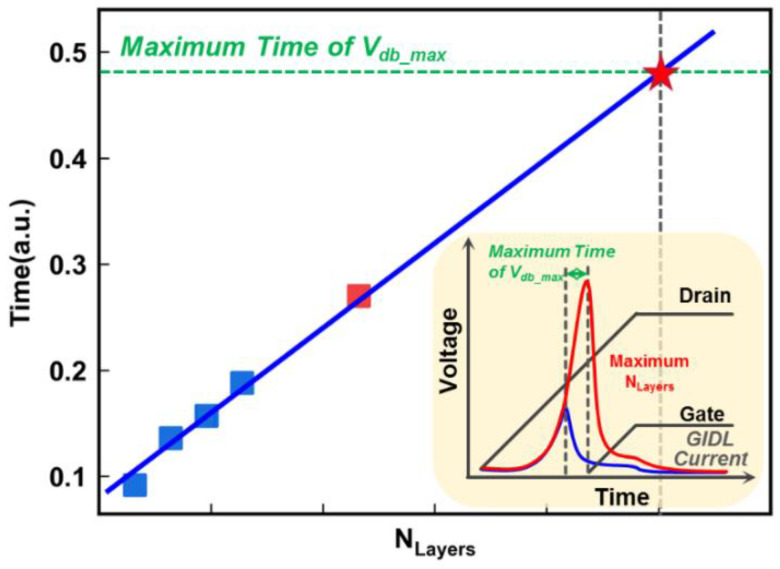
The correlation between the duration of *V*_db_max_ and the *N*_Layers_, whereby the maximum time of *V*_db_max_ determines the maximum stacking layers.

To explore the limitation of the maximum stacking layers along the NAND string of the GIDL erase operation, it is essential to determine the time when maximum *I*_gidl_ occurs to achieve a consistent channel potential at different positions. Based on the relationship between *V*_db_ and the *N*_Layers_, as the *N*_Layers_ increase, the duration of *V*_db_max_ also increases, and the subsequent *I*_gidl_ is determined by *V*_dg_. The maximum *V*_dg_ value is determined by the ramp of the GIDL transistor voltage, which affects the *V*_dg_ voltage jointly with the drain or source of the GIDL transistors. Therefore, the time when the GIDL transistor voltage increases determines the longest duration of *V*_db_max_ during the GIDL erase operation. By extracting the duration of *V*_db_max_ for lower layers and the longest duration of *V*_db_max_, a linear correlation between the duration of *V*_db_max_ and the number of layers is obtained. In order to verify the extensibility of the correlation, we simulated the GIDL erase by stacking more layers, and extracted the duration of *V*_db_max_, finding that it still follows this correlation (red square). Finally, the limited layers of the GIDL erase were obtained using the proposed method, as shown in Figure 8. The limited layers allowed for more than four times the number of layers used in the current simulation. To achieve stacking more layers in 3D NAND Flash, the GIDL erase method needs further optimization. The current GIDL erase method erases the entire channel simultaneously, that is, the synchronous GIDL erase operation. Therefore, reducing the capacitance of each erase will effectively break the limitation of the synchronous GIDL erase method. By dividing the channel into multiple regions and applying the asynchronous GIDL erase method, the stacking of more layers in 3D NAND Flash can be achieved.

## 4. Conclusions

The bit density is generally increased by stacking more layers in 3D NAND Flash. Gate-induced drain leakage (GIDL) erase is a critical enabler for the future development of 3D NAND Flash. We have investigated the correlation between GIDL erase and the number of layers based on TCAD simulation. By studying the relationship between the drain-to-body potential of GIDL transistors and increasing the number of layers, we have explained the reason for the self-adaption of the GIDL erase. The self-adaption of the GIDL erase is achieved through the dynamic control via the drain-to-body and drain-to-gate potential. As the number of layers increases, the duration of *V*_db_max_ also increases, which, in combination with increased drain-to-gate potential, enhances the GIDL current dynamically. This leads to the same potential being reached at different positions of the NAND string. Based on the correlation between the duration of *V*_db_max_ and the number of layers, we obtained the limited layers of the GIDL erase using the proposed method. The limited layers allowed are more than four times the number of layers used in the current simulation. Furthermore, by combining the novel method of dividing the channel into multi-regions using the asynchronous GIDL erase method, 3D NAND Flash can stack more layers. We expect that the GIDL erase will contribute to the further development of 3D NAND Flash.

## Data Availability

The data presented in this study are available on request from the corresponding author. The data are not publicly available due to confidentiality request.
